# Classifying organisms and artefacts by their outline shapes

**DOI:** 10.1098/rsif.2022.0493

**Published:** 2022-10-12

**Authors:** Arianna Salili-James, Anne Mackay, Emilio Rodriguez-Alvarez, Diana Rodriguez-Perez, Thomas Mannack, Timothy A. Rawlings, A. Richard Palmer, Jonathan Todd, Terhi E. Riutta, Cate Macinnis-Ng, Zhitong Han, Megan Davies, Zinnia Thorpe, Stephen Marsland, Armand M. Leroi

**Affiliations:** ^1^ Department of Mathematics, Brunel University London, Uxbridge UB8 3PH, UK; ^2^ School of Humanities, University of Auckland, Auckland 1010, New Zealand; ^3^ School of Biological Sciences, University of Auckland, Auckland 1010, New Zealand; ^4^ School of Anthropology, University of Arizona, Tucson, AZ 85721-0030, USA; ^5^ Classical Art Research Centre, Ioannou Centre for Classical and Byzantine Studies, University of Oxford, Oxford OX1 3LU, UK; ^6^ School of Science and Technology, Cape Breton University, Sydney, Nova Scotia, Canada B1P 6L2; ^7^ Department of Biological Sciences, University of Alberta, Edmonton, Alberta, Canada T6G 2E9; ^8^ Department of Earth Sciences, Natural History Museum, London SW7 5BD, UK; ^9^ Department of Life Sciences, Imperial College London, London SW7 2AZ, UK; ^10^ School of Mathematics and Statistics, Victoria University of Wellington, Wellington 6012, New Zealand; ^11^ Te Pūnaha Matatini, New Zealand

**Keywords:** classification, shape analysis, diffeomorphisms, biology, archaeology

## Abstract

We often wish to classify objects by their shapes. Indeed, the study of shapes is an important part of many scientific fields, such as evolutionary biology, structural biology, image processing and archaeology. However, mathematical shape spaces are rather complicated and nonlinear. The most widely used methods of shape analysis, geometric morphometrics, treat the shapes as sets of points. Diffeomorphic methods consider the underlying curve rather than points, but have rarely been applied to real-world problems. Using a machine classifier, we tested the ability of several of these methods to describe and classify the shapes of a variety of organic and man-made objects. We find that one method, based on square-root velocity functions (SRVFs), outperforms all others, including a standard geometric morphometric method (eigenshapes), and that it is also superior to human experts using shape alone. When the SRVF approach is constrained to take account of homologous landmarks it can accurately classify objects of very different shapes. The SRVF method identifies a shortest path between shapes, and we show that this can be used to estimate the shapes of intermediate steps in evolutionary series. Diffeomorphic shape analysis methods, we conclude, now provide practical and effective solutions to many shape description and classification problems in the natural and human sciences.

## 1. Introduction

Given a set of images of objects, we may wish to classify them by their *shapes*, by which we mean their forms stripped of any differences in size, orientation, position in space or surface patterns. Humans intuitively understand shape in this sense: we identify objects that have the same shape even when they differ in size or are oriented at different angles relative to us [1–5], and other animals seem to have similar abilities [6,7].

The analysis and classification of shapes has applications in fields as varied as biology, medicine, archaeology, image analysis and architecture [3,8,9]; many algorithmic methods that allow us to analyse shapes objectively have, accordingly, been proposed. These methods differ in how they describe shapes and estimate the distances among them. A shape outline can be represented in two fundamentally different ways: either as a set of points or as a curve. If represented as sets of corresponding points then the distance between two shapes is typically computed as the sum of the squared distances between the points; if represented as curves then as the amount of bending and stretching required to transform one into the other [10,11]. These two approaches differ not only in how they represent shapes and the distance metrics that they use; they also make different assumptions about the geometry of shape space.

We use a distance-based classifier to compare shape analysis methods based on these two shape representations. Based on the outline contours of objects from three real-world datasets we test whether the methods are comparable to human experts performing the same shape-based clustering. Follow this we consider other analyses that these methods can usefully be applied to in order to support scientists in any field where shape may provide useful information.

### 1.1. The strange geometry of shape space

Although a planar shape can be represented as a curve drawn in two-dimensional space, the space of *N* point positions representing that curve has many more dimensions: for each of the *N* points we have *x* and *y* coordinates, so need 2*N* numbers to describe each shape. Point configurations that differ only in position, scale, reflection, rotation or some combination of these, describe the same shape, which means that the set of different *shapes* is not the whole of $R^{2N}$, but a subspace of it. And that, in turn, means that shapes live not in ordinary Euclidean space, but in a nonlinear space embedded within it [12,13]. For the basic case of triangles, which are in $R^{6}$—three points in $R^{2}$—the shape space of allowable shapes is a hemisphere [2,14]. The points describing more complex shapes exist in unknown, but certainly much more complex, shape spaces. And curves, which are continuous, exist in infinite-dimensional shape spaces [15].

Geometric morphometric methods treat outline shapes as sets of points, assuming that the points identified on each shape are in correspondence and thus parametrize the shape [8,9,16]. One common approach is to treat the coordinates of each point as a statistical random variable in Euclidean space, assuming that the variations are small enough to be approximated *linearly*. Known variously as eigenshape analysis [17–19] or statistical shape models [20], they begin by extracting shapes from forms by standardizing position, scale, and rotation using a procedure called Procrustes alignment [21]. The dimensionality of the space of coordinate points is then reduced by a method such as principal components analysis (PCA), and the sum-of-squared distances between shapes computed among the vectors of derived variables.

**Figure 1.**
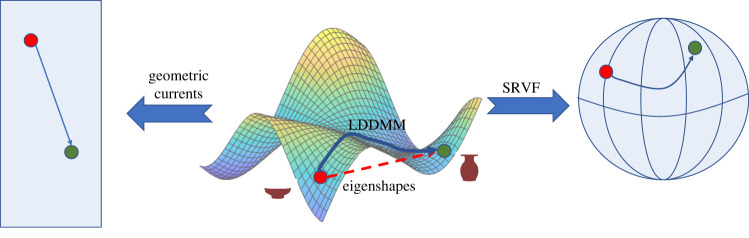
An overview of shape analysis methods. Shapes, such as the two vases shown, are described by points in a manifold of possible shapes (centre). Eigenshape analysis ignores the complex geometry of shape space and assumes that the space is Euclidean. Diffeomorphic methods, by contrast, either work on the original shape space (e.g. large deformation diffeomorphic metric mapping (LDDMM)) or else transform the original shapes into a simpler space—a sphere in the case of square-root velocity function (SRVF) or a vector space in the case of geometric currents.

The strange geometry of shape space means, however, that distances between sets of point coordinates may not be very useful as aids to classification of different shapes. An alternative is to consider the shape as a piece of elastic and ask how much ‘energy’—stretching and bending—is required to transform one shape into another; conceptually this is an extension to the thin-plate spline methods of geometric morphometrics [8]. Under certain restrictions, one curve can be continuously deformed into another using a smooth, invertible, function, i.e. a *diffeomorphism* (in contrast, thin-plate spline methods are not necessarily invertible and lose some mathematical benefits). Large deformation diffeomorphic metric mapping (LDDMM) algorithms transform shape curves into each other and estimate a distance directly in the space of diffeomorphisms, which is an infinite-dimensional manifold that can be equipped with a Riemannian metric [15,22] (figure 1). As with eigenshapes, they deal with form not shape, and so require that Procrustes alignment be applied first.

It can be beneficial to simplify the shape space by transforming the shapes before analysis. The square-root velocity function (SRVF) method maps shapes in such a way that the shape space is a sphere. The distances among shapes can, then, be easily computed as great circles [11,23,24] (figure 1). Geometric currents do something conceptually similar, transforming each shape into a mathematical function that can be represented as a point in the standard Euclidean vector space based on geometric measure theory [25]. Since this linear space is equipped with a Euclidean metric, it very easy to compute distances among shapes, and other standard statistical techniques such as PCA can also be used [26,27]. However, unlike SRVFs, it is not possible to transform the points in the new space back into the original shapes. Although the distances are now Euclidean, the geometric current transformation preserves much of the information present in the original shape space [26]. Neither the SRVF nor the geometric currents methods require Procrustes alignment.

Geometric morphometric methods have been used in fields such as evolutionary biology [8,28], medical image analysis [29,30] and archaeology [16,31,32] for many years. Diffeomorphic methods, by contrast, have been applied only recently (e.g. [33–41]). As far as we know, these two, rather different, approaches to shape analysis have not been tested against each other on real objects. Diffeomorphic methods should, in principle, provide better estimates of the true distances among real objects, but whether they do so in fact—and whether any gain in accuracy justifies their greater computational cost—is unclear. Given the range of shape variation among real objects, the approximation that shape space is linear may even be reasonable [14].

Here, then, we test one approach of geometric morphometrics, semi-landmarks eigenshapes analysis, and three diffeomorphic methods—LDDMM, SRVF and geometric currents—against each other in order to find out which of them performs best when classifying the shapes of real objects. The objects belong to three very different classes—ancient Greek vases, the leaves of Swedish trees and gastropod shells—chosen so that our results can be directly applicable to support archaeologists, botanists and zoologists, all of whom describe the shapes of the things that they study.

Each of our datasets is divided into classes, for example, genera of shells. Our test, then, rests on the ability of a statistical classifier, trained on distances computed by our various methods, to identify those classes. We show that, for all datasets, one diffeomorphic method—that based on SRVFs—is superior to all other methods, including eigenshapes which, however, usually works impressively well. However, we also wanted to know what a good classification—the kind that a trained human might make—looks like, so we asked experts to undertake the same test. We find that most of our algorithmic methods beat the human experts. We conclude that such methods, particularly those that operate on curves rather than points, are very effective when applied to many shape classification problems, and can even be superior to humans. Finally, in homage to the grandfather of shape analysis, D’Arcy Wentworth Thompson, we show that some of these methods provide an answer to the problem that he posed in Chapter XVII of *On growth and form* [42]: how mathematics might be used to transform one shape into another.

## 2. Results

**Figure 2.**
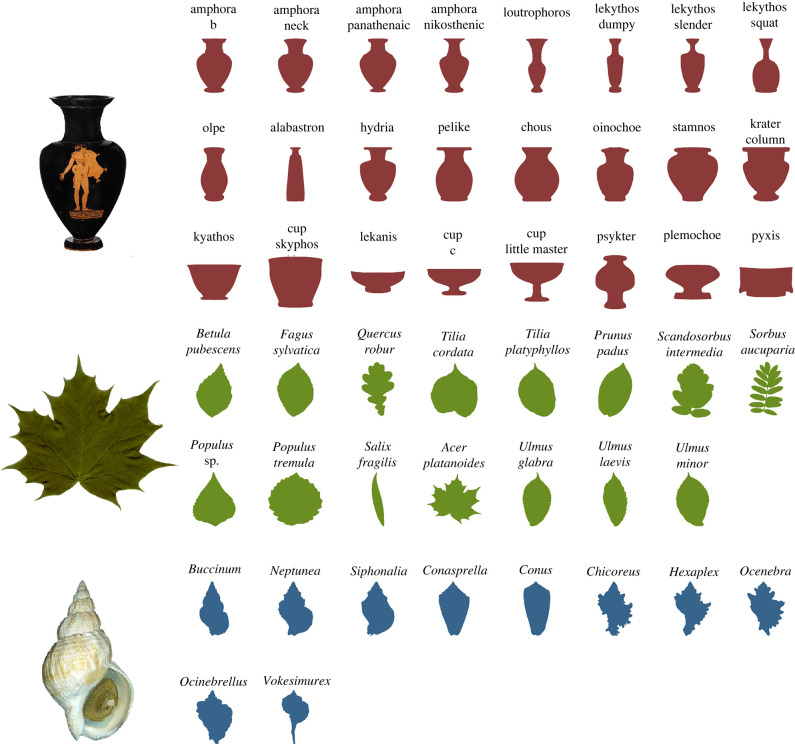
Shape diversity in, from top to bottom, ancient Greek vases, Swedish leaves and gastropod shells. *Left*: An example of an original image for each class; *right*: filled outlines of a randomly chosen member of each class within the three datasets.

We studied the two-dimensional outline shapes of three very different sets of objects: vases, leaves and shells. The vase outlines are based on 716 images of Athenian black- or red-figure vases classified into 24 classes: the shape categories used by vase scholars; the leaf outlines are based on 440 images of Swedish leaves classified into 15 Linnaean species; the shell outlines are based on 235 images of gastropod shells classified into 10 Linnaean genera. Figure 2 shows, for each dataset, one of the original images from which outlines were extracted, as well as the outline of a randomly chosen member of each class. Our examples embrace a great variety of shapes. Where the outlines of Greek vases are mostly smooth, those of shells and leaves are often very jagged; and while our shells have quite similar aspect ratios, some leaves are needles, others are pancake-like and others are something between. Within each class, the individual objects are unique and distributed more-or-less evenly among classes.

### 2.1. Square-root velocity functions are superior to other shape description methods

To test our four shape description methods—eigenshapes, LDDMM, SRVFs and geometric currents—we first calculated the pairwise distances between all objects of each set—vases, leaves and shells—using each method. We then trained a statistical classifier on the distances among a training set of between 51% and 67% of the objects, and then asked the classifier to assign the remaining test objects to a class based on the distances among the objects. To ensure that our results did not depend on the chance allocation of individuals among training and test sets, we constructed a hundred different sets by random stratified sampling and ran the classifier on each. Since the shape analysis methods compute distances between shapes, the obvious classifier is one that uses such distances directly, here the *k*-nearest neighbour (*k*-NN) classifier. We measured classification success as the *F*_1_-score, the harmonic mean of precision and recall of the obtained classification relative to ground truth [43]. Note that *k* in *k*-NN is a user-specified parameter that selects the number of neighbours to use; we selected an optimal value for *k* for each shape method and dataset in initial experiments based on the *F*_1_-score.

**Figure 3.**
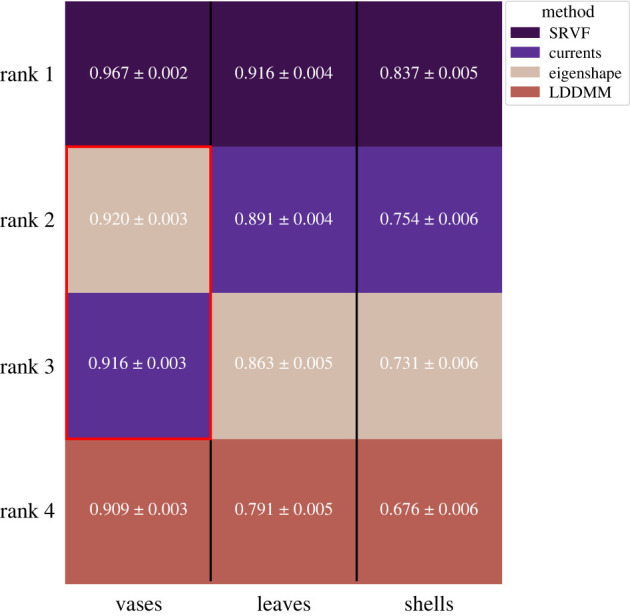
Machine classification of three sets of objects using four shape analysis methods. Our measure of performance is the *F*_1_-score, which captures the congruence between the *k*-NN classification and ground truth. The mean *F*_1_-scores ± 95% confidence intervals are based on randomly sampled training/test sets. The methods are ranked by decreasing *F*_1_-scores within each object type. All methods showed statistically significant differences in performance from each other (*P* < 10^−6^, two-tailed *t*-tests) except eigenshapes and geometric currents for vases, as indicated by a red box (*P* = 0.1). Each shape analysis methods has tuning parameters and, for each method and class of objects, we tried many combinations of them; only the results for the best-performing combination of parameter values on each object class are shown. The best method in all cases is the one based on SRVFs, followed by geometric currents, but eigenshapes perform better than LDDMM.

Even though the training sets were small—a few hundred individuals divided among 10–24 classes—the *k*-NN proved remarkably good at classifying outline shapes. Its ability to do so, however, depended on the shape description method used. Figure 3 shows the ranked performance of each method over the object samples. The SRVF method was the top-ranked method in all cases, being able to classify vases into their classes with 97% accuracy, leaves with 92% and shells with 84% (*F*_1_-scores); geometric currents performed next best overall, followed by eigenshapes.

In order to show each method to its best advantage we varied their parameters (see §4); figure 3 reports the best result for each class. The variation in performance that comes from tweaking parameters can be instructive. When trying eigenshapes, for example, we varied the number of principal components that went into the distances and found that, in all cases, the winner used at least 90% of the total variance and for vases 99.9%, which suggests that some of the shape differences between classes are very subtle indeed.

**Figure 4.**
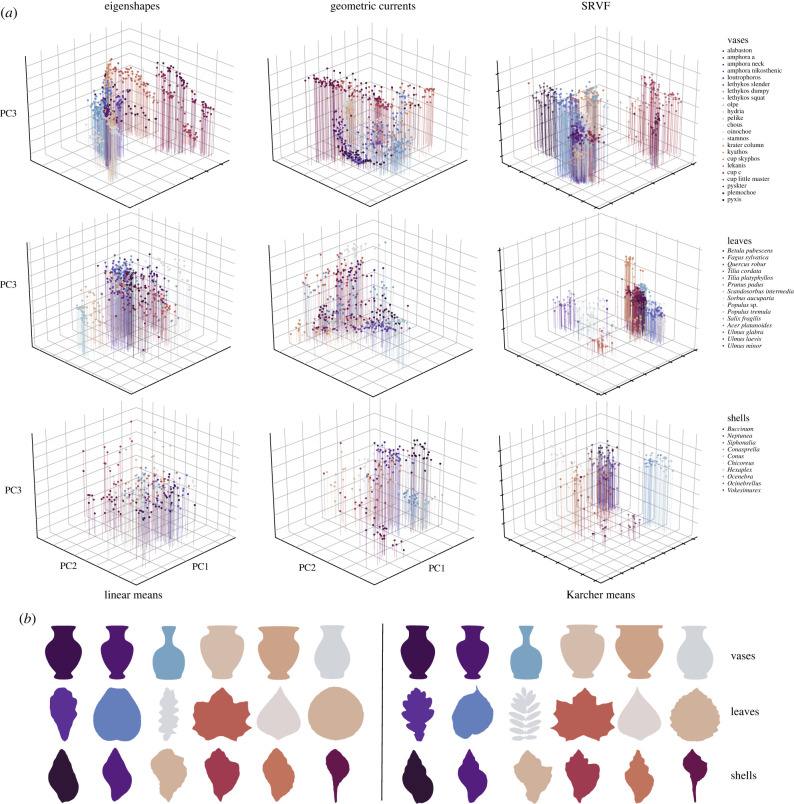
(*a*) Visualizing the positions of objects in shape space. Objects plotted in the first three principal components of: *left* eigenshapes, *centre* geometric currents and *right* SRVF space. Classes are colour-coded within each object type: *top* vases, *centre* leaves, *bottom* shells. In general, the classes are increasingly well separated moving from eigenshapes to geometric currents to SRVF space. (*b*) Mean shapes for selected classes of objects. *Left*: linear means from eigenshapes; *right*: Karcher means from SRVFs. The Karcher means usually look more like the outlines of individual objects than the linear means do, the gain in detail being particularly pronounced for complex leaf and shell shapes.

The best method, using SRVFs, improves shape classification accuracy over eigenshapes by 5–10% depending on the object class. However, the superiority of diffeomorphic methods is also evident when we plot the positions of the objects in the relevant shape space. Eigenshapes, geometric currents and SRVFs all yield principal components and, in general, the classes are better separated in principal component spaces based on geometric currents or SRVFs than they are in eigenshape space (figure 4*a*).

We can also compute the average shape of each class using our various methods. For SRVFs this is the Karcher (Fréchet) mean, an average shape estimated in Riemannian space [44]. Figure 4*b* shows that, where eigenshape means are rather amorphous, even blob-like, Karcher means retain more detail and so resemble the original objects much more closely (compare the objects in figure 4*b* with those in figure 2). Thus our results show that the diffeomorphic shape description method based on SRVFs is better than the standard method, eigenshapes, at classifying the shapes of real objects and also at producing accurate averages of groups of objects. We note, however, that eigenshapes actually work surprisingly well and beat one diffeomorphic method, LDDMM, at least in our implementation.

**Figure 5.**
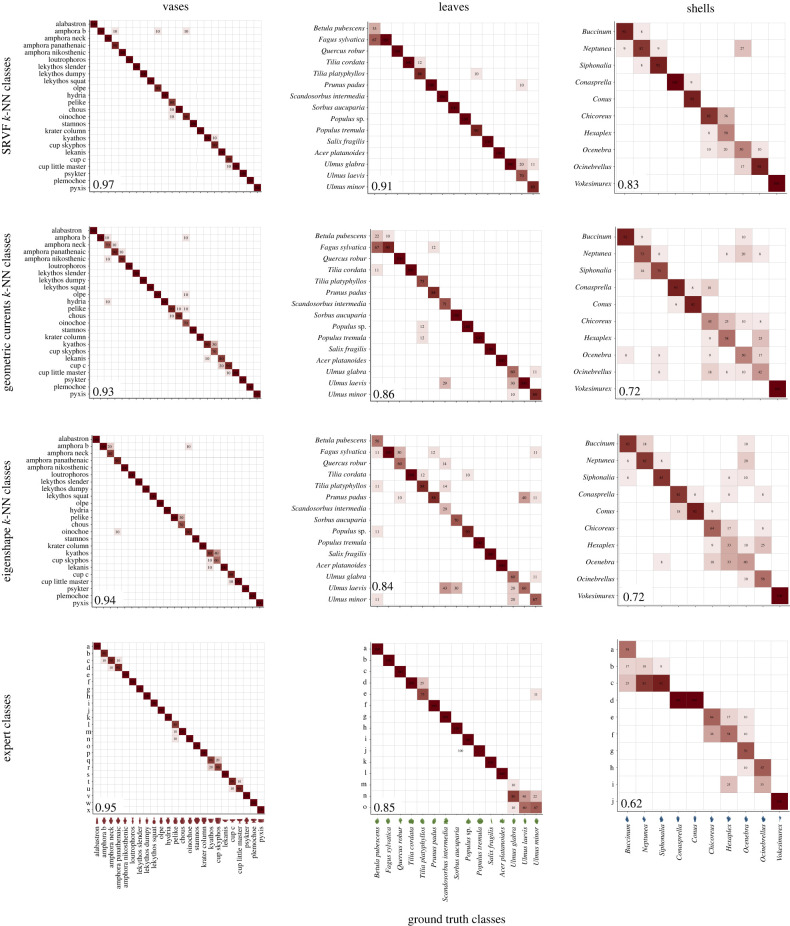
Confusion matrices showing the ability of various methods to classify three classes of objects. Were the objects all perfectly classified, all elements would be placed on the diagonal, so off-diagonal elements are mis-classifications. The *F*_1_-score of each classification is given in the bottom left corner of each panel. Top to bottom: classification by SRVF *k*-NN, geometric currents *k*-NN, eigenshapes *k*-NN, the best of the three experts for each dataset; left to right: classification of vases, leaves, shells. Note that the experts were asked to place outlines into similar shape groups, but not to label the groups.

Why are some of our diffeomorphic methods superior to geometric morphometrics when used in a supervised classifier? It may be supposed that the curvature of shape space only matters when comparing objects of very different shapes and that, when comparing similar objects, a linear approximation will do. However, this is clearly not so. As the confusion matrices show (figure 5), the superiority of SRVFs over eigenshape distances lies precisely in their ability to discriminate among very similar objects, such as the two types of stemless cups (kyathos and skyphos), the three genera of Buccinid shells (*Buccinum*, *Neptunea* and *Siphonalia*), or the two species of *Ulmus* leaves. This suggests that shape space is geometrically complex at even very small scales.

### 2.2. A machine shape-based classifier is superior to human experts

We would like a machine classifier that classifies at least as well as humans do. But not all humans are equally adept at classifying all things. To find out how well our shape-based *k*-NNs perform we formed a panel of experts composed of three classical vase scholars, three botanists and three malacologists, and asked them classify the dataset of which they were knowledgeable into classes (i.e. the malacologists only got shells). We generated a test set of outlines that resemble those in figure 2 and asked all three experts and the SRVF-based *k*-NN to cluster them into a specified number of classes, without naming those classes. Thus the role of experts and *k*-NN were the same except that, instead of being based on a training set, the experts had to rely on what they already knew.

For these particular test sets, the SRVF-based *k*-NN classifier achieved *F*_1_-scores of 0.971, 0.908 and 0.848, for vases, leaves and shells, respectively: comparable to the scores we found on our hundred-replicate data sets (figure 3). Our experts were not as good: the mean *F*_1_-scores of the three (±1 s.d.) were 0.847 ± 0.087, 0.799 ± 0.039 and 0.574 ± 0.044 for the same objects. The best that any expert did on any dataset was 0.95 (for vases), but even that expert was beaten by the machine. Interestingly, the rank order of the average abilities of our expert groups—vase scholars > botanists > malacologists—is the same as that of the machine classifiers, which suggests that the *a priori* taxonomies of vases, leaves and shells that we used embody successively less shape information. Moreover, as the confusion matrices show, experts and algorithms tend to make the same kind of mistakes (figure 5). Where our experts tended to confuse kyathoi and skyphoi vases, the three species of *Ulmus* leaves, and shells belonging to the muricid genera *Hexaplex* and *Chicoreus*, so did the algorithms. There are some differences. The SRVF-based *k*-NN correctly classified most *Conus* and *Conasprella* shells correctly even though they have very similar cone-shaped shells. Our experts, by contrast, all failed to do so. In general, however, our results suggest that, when classifying shapes, human experts and machine classifiers based on distances in shape space do much the same thing. It is just that algorithms do it better.

### 2.3. Classifying in global shape space

**Figure 6.**
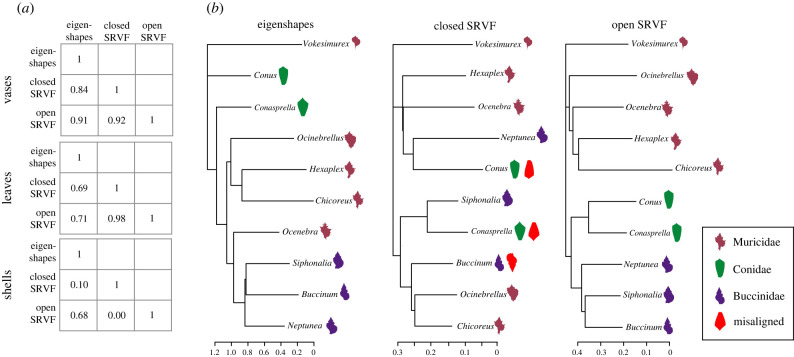
(*a*) Pearson correlation coefficients (*r*) between pairwise class average distances based on three methods: eigenshapes, closed-curve SRVFs and open-curve SRVFs. All correlations for vases and leaves are large, implying that the relative positions of the classes are similar regardless of metric, but for shells the correlations involving closed-curve SRVFs are very small, implying that this method is estimating distances in a very different way from the other two. (*b*) Neighbour joining trees using the three sets of distance metrics among groups; *Vokesimurex* was arbitrarily chosen as the root for all trees. With some exceptions the eigenshape distances tree (left) groups the genera roughly into families; the closed-curve SRVF tree (centre), however, does not do so at all. This is probably because the algorithm misaligned some curves relative to their biological orientation; several possible misalignments (rotations) are illustrated by red shells. A tree based on open-curve SRVFs constrained to have homologous start and end landmarks (right) groups the genera into monophyletic families and therefore captures the evolutionary history of these gastropod snails best of all.

As discussed above, SRVF distances are better than eigenshape distances at discriminating very similar shapes. But it does not follow from this that they are better at classifying very different shapes. To investigate this, we began by examining the relative positions of our object classes in shape space when measured by either eigenshape or SRVF distances. We estimated the average pairwise distances among all classes within a group (e.g. vases) for both distance metrics and found that, for leaves and vases, the Pearson correlation between the two distance metrics was high (*r* = 0.69 and 0.84, respectively), which implies that the relative distances of object classes are similar regardless of the metric used. Shells, however, were very different: the two distances were essentially uncorrelated (*r* = 0.1). One of the methods must be locating classes of shells incorrectly in shape space (figure 6*a*).

To find out which method was at fault, we constructed neighbour joining trees using the average distances between shell classes (figure 6*b*). Since the classes are genera, these trees can be viewed as phylogenies. Based only on shape we do not expect their topologies to be the same as a DNA-based phylogeny, but we do expect genera that belong to the same family to group together. This was roughly true for eigenshapes, but not SRVF distances. For example, the Conid genera, *Conus* and *Conasprella*, have very similar shapes—our experts could scarcely tell them apart—and are very different from all the other genera, yet the SRVF tree splits them apart and makes them sister taxa to different Buccinids (figure 6*b*, ‘closed SRVF’). SRVF distances, then, although good at discriminating among very similar genera, make no biological sense when considering shell space as a whole.

We hypothesized that this failure is due to the fact that the SRVF algorithm knows nothing about biological homology, and can rotate the objects. When finding the shortest path between two curves the algorithm does so even if that means matching the apex of one shell to the siphonal canal of the other. Misalignments of this sort presumably occurred repeatedly (figure 6*b*, ‘closed SRVF’, red shells), and so we incorporated an implementation of the SRVF-based algorithm that uses open, rather than closed, curves and constrained it so that the first and last coordinates, which are based on homologous points near the apices, were matched to each other. Thus, the SRVF-based algorithm now makes use of two landmarks at the start and end of the curve (conceptually, the eigenshapes algorithm uses all the points as landmarks, but may optimize their locations). We found that using these ‘open’ curves, the correlation between the eigenshapes and SRVF distances improved somewhat for vases and leaves and considerably for shells: where previously it was *r* = 0.1 now it was *r* = 0.68. Moreover, the NJ tree based on the open-curve SRVF distances was far superior to either eigenshapes or closed-curve SRVF distances, for now all three families—Muricidae, Conidae and Buccinidae—were monophyletic (figure 6*b*, ‘open SRVF’). The open-curve SRVFs were also at least as good as the closed-curve SRVFs on the original *k*-NN classification task (*F*_1_-scores: 0.969 ± 0.002, 0.938 ± 0.003 and 0.844 ± 0.006 for vases, leaves and shells respectively). Thus, when classifying objects of very different shapes we recommend ensuring that the objects are parametrized using the same start and end points, and restricting the SRVF algorithm to match open curves with constrained start and end points, as well as potentially incorporating other homologous landmarks if provided.

### 2.4. Finding the shortest paths in shape space

**Figure 7.**
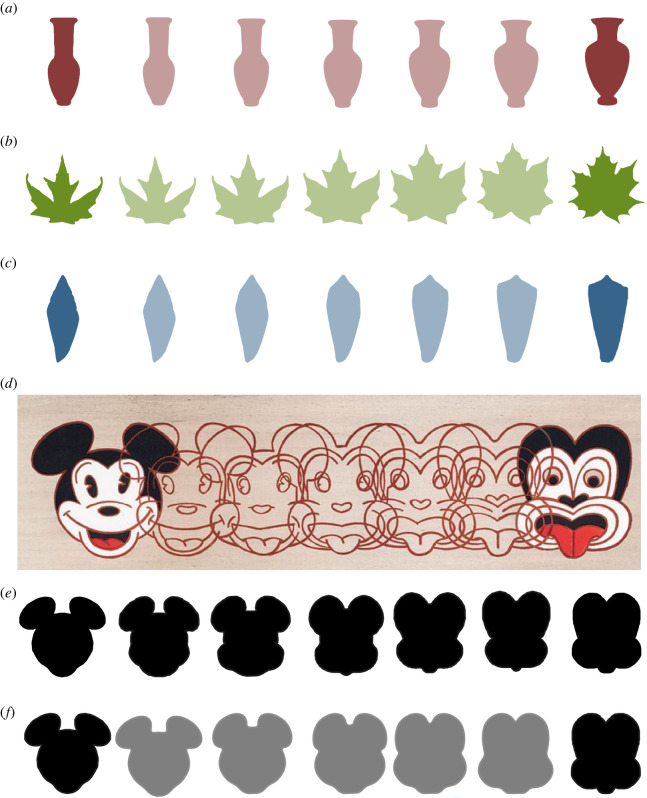
Examples of shape transformations along geodesic paths. (*a*) A Proto-Attic Neck Amphora (675–700 BCE) transformed in five steps into an Athenian Red Figure Neck Amphora (525–475 BCE); (*b*) an Early Miocene (20–18 Ma) maple leaf *Acer palaeosaccharinum* into the recent *Acer platanoides*; (*c*) the Late Paleocene *Hemiconus leroyi* (59.2–56 Ma) into the recent *Conus furvus*; (*d*) *Mickey to Tiki Tu Meke (1995)* by New Zealand artist Dick Frizzell; (*e*) Frizzell’s shapes isolated as outlines; (*f*) Mickey into Tiki outlines as the shortest distance in SRVF shape space.

The SRVF algorithm and LDDMM work by transforming shapes into each other. When doing so, they find a geodesic—the shortest path in shape space. Any point along this path can be back-transformed into a shape in the original space to produce a transformational series. To illustrate this we transformed the outline of a plausible ancestor, or at least ancient relative, to one of our modern objects and inferred some intermediates. Figure 7*a*–*c* shows the transformation of a Proto-Attic Neck Amphora (725–675 BCE) into an Athenian Red Figure Neck Amphora (525–475 BCE) [45]; an Early Miocene (20–18 Ma) maple *Acer palaeosaccharinum* [46] into the recent *A. platanoides*; and the first known Conid gastropod, the Late Paleocene *Hemiconus leroyi* (59.2–56 Ma) into the recent *Conus furvus* [47,48]. These examples are only illustrative: we do not claim that the earlier objects are true ancestors of the more recent ones. Indeed, the transformed objects need not be linked by evolutionary descent at all. In 1995, the New Zealand Pop artist Dick Frizzell transformed an American icon, Mickey Mouse, into a Māori one, the Tiki (figure 7*d,e*; used with permission of the artist). The SRVF geodesic path from Mickey to Tiki is slightly different from the artist’s—and 23% more efficient (figure 7*f*).

## 3. Discussion

Although geometric morphometric methods have been widely used, they only approximate distances in the complex geometry of shape space [13,14]. For biological objects this approximation largely suffices [14] and, consistent with this claim, we find that eigenshapes analysis generally performs quite well at our classification task. However, two diffeomorphic methods, based on SRVFs and geometric currents, are even better at distinguishing and classifying objects of different shape, and this is true for objects as disparate as vases, leaves and shells. In addition, the mean shape of groups of objects in these shape spaces also clearly preserve more detail than linear shape means do. These results imply that diffeomorphic methods, until now mostly studied by mathematicians, belong in the scientist’s toolbox. All the implementations that we used are publicly available (see Material and methods).

The three diffeomorphic methods do not perform equally well at the classification task. Since LDDMM does not simplify the shape space it might be expected that it would give the most accurate distances relative to human experts. In fact, of the three it performs the worst. This is because the metric used trades-off the precision of the transformation with the length of the path between them. For this reason, it sometimes finds a transformation that is only close to the true target shape, and so may miss some of the finer distinctions among our classes. Since SRVFs and geometric currents induce spaces with much simpler geometries, they should be able to match curves exactly. However, implementation in a computer requires additional constraints. The geometric currents algorithm first discretizes the shapes and, in doing so, sacrifices some information about them, while the SRVF algorithm avoids this at the cost of simplifying the path between the shapes. *A priori* it is not clear which of these approaches would be most effective, but empirically the SRVF approach is for these datsets.

Our machine shape classifier worked best on the vase dataset, slightly less well on leaves and only moderately well on shells. It may be supposed that these differences in performance depend on the shapes themselves. However, since the performance of the experts showed exactly the same rank order, it is much more likely that they depend on the quality of the classes. While our ground-truth classes were imposed by humans and chosen by us in the expectation that their members have, on average, different shapes, their natures vary. The vase classes are based on a scholarly taxonomy that largely depends on their gross shapes, but the leaf and shell classes are not; for modern biological genera and species are distinguished not only by gross shape and positioning of constituent parts (e.g. spiral ribs, varices, leaf veins), but also microscopic, ecological, behavioural and genetic traits or abstract properties such as the ability to interbreed. Even the differences between vase classes are not all visible from their outlines, depending, in part, on constructional details. This means that the amount of information about class identity that is visible from shape outlines varies greatly among the three datasets.

Our classifiers used only shape rather than the many other features that might distinguish these groups. Furthermore, the classifier that we used—a *k*-NN—requires very small training sets compared to the large training sets required by more sophisticated ML methods such as a convolutional neural network (CNN). However, *k*-NN is the natural choice, since our analysis gives distances among shapes rather than features. Even so, the success of our shape-based classification is remarkable. We imagine that they might be useful for the automatic classification of the innumerable objects that differ in shape, not only those we have studied here, but even things as diverse as protein structures, the spectrograms of bird songs or the melodies of pop songs (e.g. [49–52]). Given suitable data our methods could also be applied to three-dimensional shapes (e.g. [32,53]); see [54] for a three-dimensional generalization of the SRVF. This would be particularly useful for rotationally asymmetrical objects, but also require much more computational effort.

Our classifier was more accurate than the judgements of experts, almost regardless of the shape-analysis method under the hood. Why is this? We asked our experts and found that they were often led astray by prior knowledge. Where the machine classifier was trained to distinguish the groups actually present, the experts sometimes sought the groups that they thought *should* have been there. For example, all three malacologists failed to distinguish between the closely related genera *Conus* and *Conasprella*. They did so because the classification of the Conidae remains unsettled [55], and the relationship between shell shape and genera unclear. Indeed, one of our experts had second thoughts about the cones, gave us a revised classification before being told the ground truth, and got the best *F*_1_-score among the malacologists, 0.716. Our intention is not to diminish experts who, after all, usually have much more information about the objects that they classify, but rather show how effective machine shape-classifiers can be, even when based on very small training sets. Had we given our experts information about the particular classes that we had pre-determined—either as verbal descriptions or examplars—then the results may well have been different.

Compared to linear methods, diffeomorphic approaches are computationally expensive. In the implementations we used, a SRVF-based or LDDMM registration for a single pair of shape outlines takes, on average, 1–3 s to process on a modern laptop. Computing all 255 970 pairwise distances for our Greek vase dataset of 716 objects takes, then, 85 h if performed sequentially. The geometric currents algorithm is much faster and takes about 10 s to complete the same task, although it does suffer from some memory issues. However, eigenshapes—Procrustes alignment, PCA and distance calculations—takes, on average, only 1.5 s (all timings based on the same core i7 computer and based on Python code).

A fundamental difference between the methods is that geometric morphometric methods depend on the correspondence of explicitly homologous points—landmarks—while the diffeomorphic methods use the underlying curve outline. We initially used closed curves, that is, curves without a start or end. This is beneficial if the objects are presented in any orientation, but, as we have shown, it can provide too much variability. When diffeomorphic algorithms rotate shapes relative to each other to find lower energy paths they should, in general, align homologous parts to each other. However, if the shapes are sufficiently different they need not: instead, the spire of one shell might be aligned to another’s siphonal canal or the neck of one vase to another’s base. Indeed, when comparing distantly related classes (particularly within the shells, but also sometimes the vases) we found instances where this appeared to be the case. Such misalignments may not matter for the purposes of local classification, but any evolutionary interpretation of the distances would be incorrect, for the inferred path would be one that evolution could not possibly have taken.

If the shapes are pre-processed to incorporate some information about homology then this can be used. To deal with this case, we constrained the SRVFs to be based on open curves with fixed start and end points, which removes the ability of the algorithm to rotate the objects (a similar approach is taken in [56]). The same methodology could be used to incorporate other homologous landmarks should they exist, meaning that the *a priori* information established over many years of scholarly study can still be incorporated. However, our results show that the diffeomorphic methods are applicable even when this information is not available. Of course, as with any classification method, verification and further analysis of the results require expert human input.

More than a hundred years ago, D’Arcy Wentworth Thompson posited his ‘theory of transformations’, which held that species closely related by evolutionary descent should also be related by ‘simple’ shape transformations; and that ‘small’ transformations indicate particularly close evolutionary affinities [42,57]. To demonstrate this Thompson relied on outline drawings of an animal, adding a rectilinear grid that was deformed using a regular transformation, with the image of the animal deformed along with it until it more closely matched another animal. Our modern equivalents dispense with the grid and match the curves more accurately. However, in spirit they are the same, and our transformations illustrate how the evolution of shape in Riemannian space can be modelled so that it might be mapped onto a phylogeny, or even used to infer one [58,59].

## 4. Material and methods

### 4.1. Datasets

The vase images were obtained from the Beazley Archive Pottery Database (BAPD; https://www.beazley.ox.ac.uk/index.htm) at Oxford University; their taxonomy, which was modified slightly from the standard shape taxonomy given in the BAPD, was checked by two experts, T.M. and D.R.-P. The leaf images are based on the Swedish Leaf Dataset at https://www.cvl.isy.liu.se/en/research/datasets/swedish-leaf/ previously used in the image analysis and shape literature [60]; the images came with species labels, which were checked by an expert, T.E.R. The shell images were obtained from Gastropods.com (http://gastropods.com); the images came with species labels whose taxonomy was standardized to the World Register of Marine Species (WoRMs; http://www.marinespecies.org) and checked by A.M.L. Each image represents a unique object and was checked to ensure that it was complete and in standard orientation. The sources of the original images are given in the Readme in this repository: https://github.com/smarsland/ClassifyingOrganismsArtefactsShape.

### 4.2. Data preparation

Shape methods require an outline of the object, and often it is necessary to extract this from a digital photograph. While this has been an area of research interest for a long time in computer vision—and is something that humans do easily—there do not yet exist completely reliable methods [61]. We used a common contour extraction algorithm, the marching squares method [62] on a binarized version of each image, with the threshold chosen experimentally. For the leaves and shells no other pre-processing was performed, but for the vases the handles were removed using a spline fit, which was manually verified and, if necessary, corrected. The vases were also made to have a reflective symmetry through a central vertical axis by computing the outline contour of each side, and using the shorter one of the pair, reflecting it to make the full shape. This removes structures such as the spouts of pouring vessels.

Each outline curve was sampled to have an identical number of equally spaced points—139 for the vases, 150 for the shells and 200 for the leaves—by sampling a cubic spline fitted to the curve. Preliminary experiments showed that at these resolutions no difference between the interpolated curve and the original shape were visible to the naked eye. The point sets were aligned using Procrustes alignment to remove the global transformations of scale, rotation and translation from the curves. This is necessary for eigenshapes and LDDMM, but not for the methods based on SRVFs or geometric currents. Examples of the resulting shape outlines with filled interiors are shown in figure 2. The same datasets of shape outlines were used when testing all methods. The shape outline data are available at the following DOIs. Vases: https://doi.org/10.6084/m9.figshare.14551002, leaves: https://doi.org/10.6084/m9.figshare.14551005, shells: https://doi.org/10.6084/m9.figshare.14551044.

### 4.3. Estimating distances

Parameters for each method were chosen experimentally based on the training data, and the upper-triangular distance matrix between all pairs of shapes computed for each method. *Eigenshapes*: We used the points that parametrize the curve as semi-landmarks. We experimented with optimizing the position of these landmarks, but it was computationally expensive and did not improve the results. We computed the principal components of the point coordinates of all shapes and, from these, the Euclidean distances among them using the first *d*-dimensions, where *d* was chosen based on the amount of the variance explained, ranging from 0.75 to 0.999. *LDDMM*: We used the implementation described in [63] available at https://github.com/tonyshardlow/reg_sde, running for 20 timesteps. *SRVF*: We used the fdasrsf library available at https://github.com/jdtuck/fdasrsf_python. For closed curves, we used the path-straightening algorithm described in [64] and available in the fdasrsf library. The algorithm transforms one shape to another in *κ* ≥ 2 steps. The output is the geodesic distance, which is the inner product in SRVF space between the first shape and the final shape in the transformation. To compute our distance matrix, we set *κ* = 2. For open curves, the points were presented in pre-defined order, and these points cannot be translated; this removes the rotations. The geodesic distances between open curves were computed using the default parameters in the fdasrsf library. This computation includes an additional re-sampling step, where curves were interpolated at 100 points using a univariate spline fit. *Geometric currents*: We used the method described by Benn *et al*. [26] available at https://github.com/olivierverdier/femshape. This implementation takes three parameters: a non-negative integer, *s*, determining the size of the matrix representation; the mesh-size, *m*; and a scaling parameter, *σ* ≥ 0. We tested three options for each parameter where 1 ≤ *s*, *σ* ≤ 4 and 16 ≤ *m* ≤ 24.

### 4.4. Machine classification

Most machine learning algorithms take as input features of the elements of the dataset (or their complete representation), rather than distances. We, however, used our various shape analysis methods to compute distances among objects and wish to classify on those. For this reason, we implemented our own *k*-NN classifier that takes a distance matrix as its input and assigns elements of the test set to the class of the majority of the closest *k* points in the training set, where *k* is a user-selected parameter. We tested values of *k* between 3 and 12 for each method and object class and found the *k* that results in the highest *F*_1_-score. We ran the *k*-NN on 100 randomly selected samples from the training sets of each dataset and computed the *F*_1_-scores. For vases the ratio of training : test set was 480 : 236, leaves 300 : 140, and shells 120 : 115. In order to ensure that training set size was not the reason for better performance in the case of vases, we also reduced the size of the training set in that case (to 10 in each class), leaving the test set alone, without significantly changing the results. Interestingly, even when reducing the training set further, to 2 in each class, the classifier still did well. We used the sklearn implementation of the *F*_1_-score with the *average* parameter set to ‘weighted’.

### 4.5. Expert classification

Each expert was given a standard test set of shape outlines as individual images and asked to partition them into *n* groups, where *n* is the number of ground-truth classes, by sorting them into folders. The objects were anonymized so that no expert had any information about them that the machine classifier did not. The experts were not asked to identify the groups that they formed. Each expert’s classification was then compared to the ground-truth classification with the *F*_1_-score.

### 4.6. Transformations

To create the transformation plots seen in figure 7, we used the SRVF path-straightening algorithm with *κ* = 5. Note that the transformations are not necessarily symmetric even if the shapes themselves are, such as Mickey and Tiki. Therefore, to display a symmetric transformation between Mickey and Tiki, we split the outlines in half and transformed these halves from one to another. The transformations were then reflected and attached. To test the efficiency of our transformation with the artist’s, albeit in a metaphorical sense, we computed the sum of the distances between consecutive outlines, i.e. the energy needed to deform one shape into the other.

## Data Availability

Sources are provided in the paper, and code is available on github as specified in the paper. Data access is described in the Material and methods.
